# Supplementary material for: Disease-associated loci share properties with response eQTLs under common environmental exposures
Source: Res Sq. 2025 May 14:rs.3.rs-6561377. Preprint. [Version 1] doi: 10.21203/rs.3.rs-6561377/v1 (PMC12136233; doi:10.21203/rs.3.rs-6561377/v1)
Supplement: 1 [file NIHPPRS6561377V1-supplement-1.pdf]

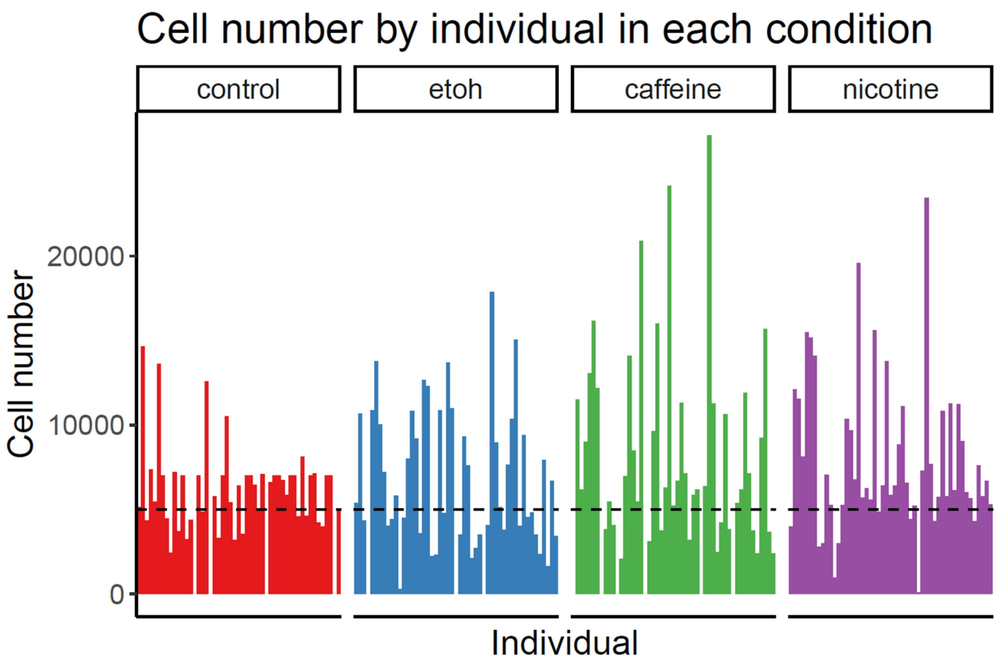

Figure S1. Summary of cell number in each individual line and condition.

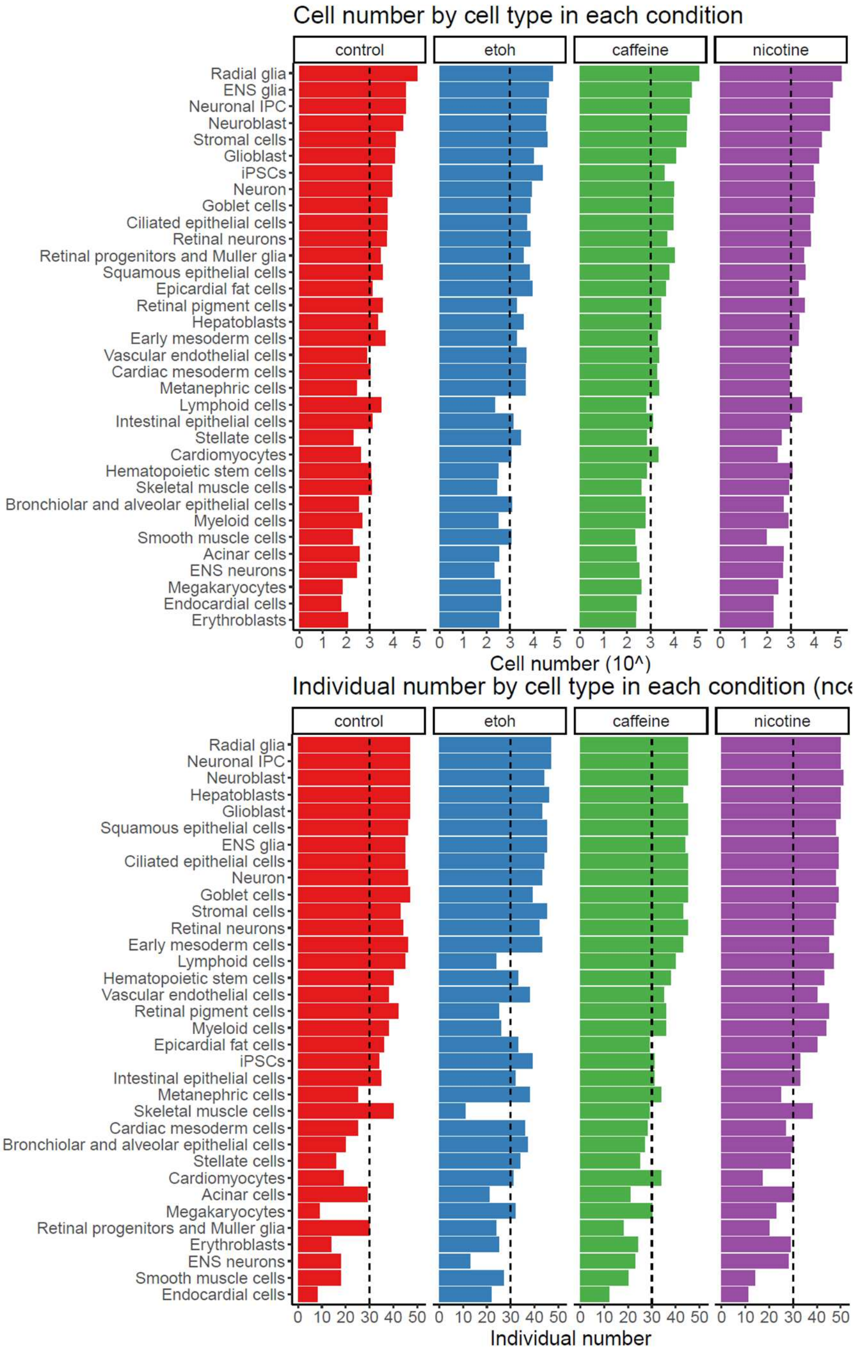

Figure S2. Summary of cell number and individual number in each cell type and condition.

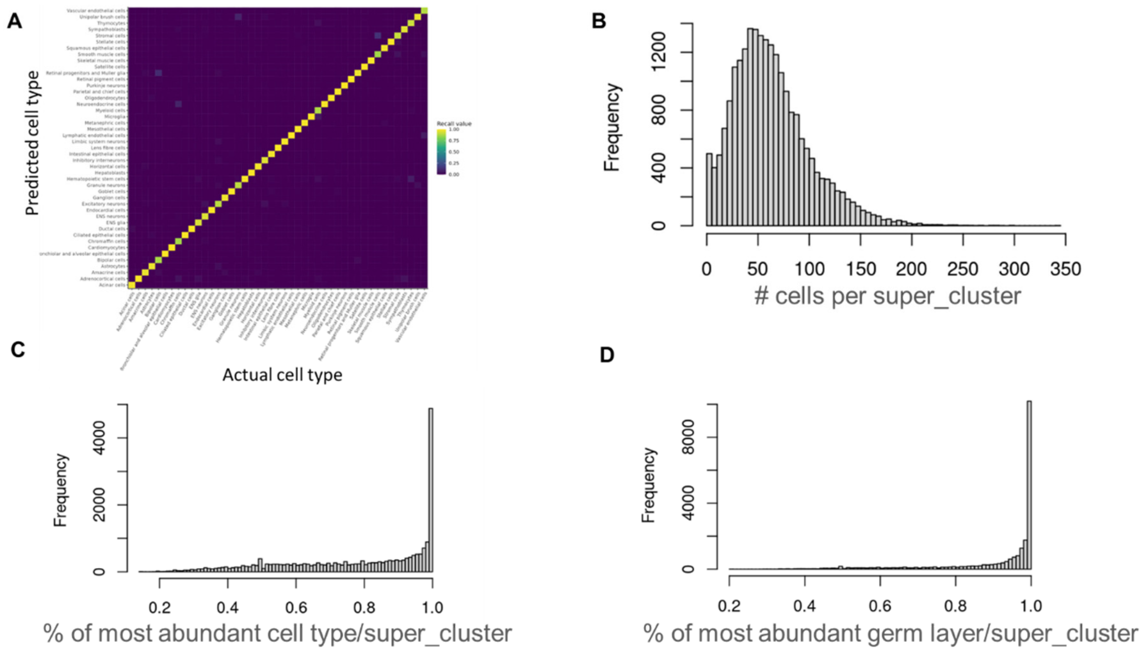

Figure S3. Validation of cell type annotation approach and results.
(A) Confusion matrix for intradataset cell type cross-validation. We split the fetal cell atlas into training and test sets where we used the training set to generate a reference marker list and the test set for cell type prediction.
(B) Distribution of cell number across 22K super clusters generated by unsupervised clustering with a high resolution, with an average of 60 cells per cluster across 1.4M cells in our study.
(C-D) Histogram of the proportion of most abundant assigned cell type (C) or germ layer (D) in each super cluster. Cells in the majority of super clusters have a homogenous (>80%) cell type/germ layer assignment.

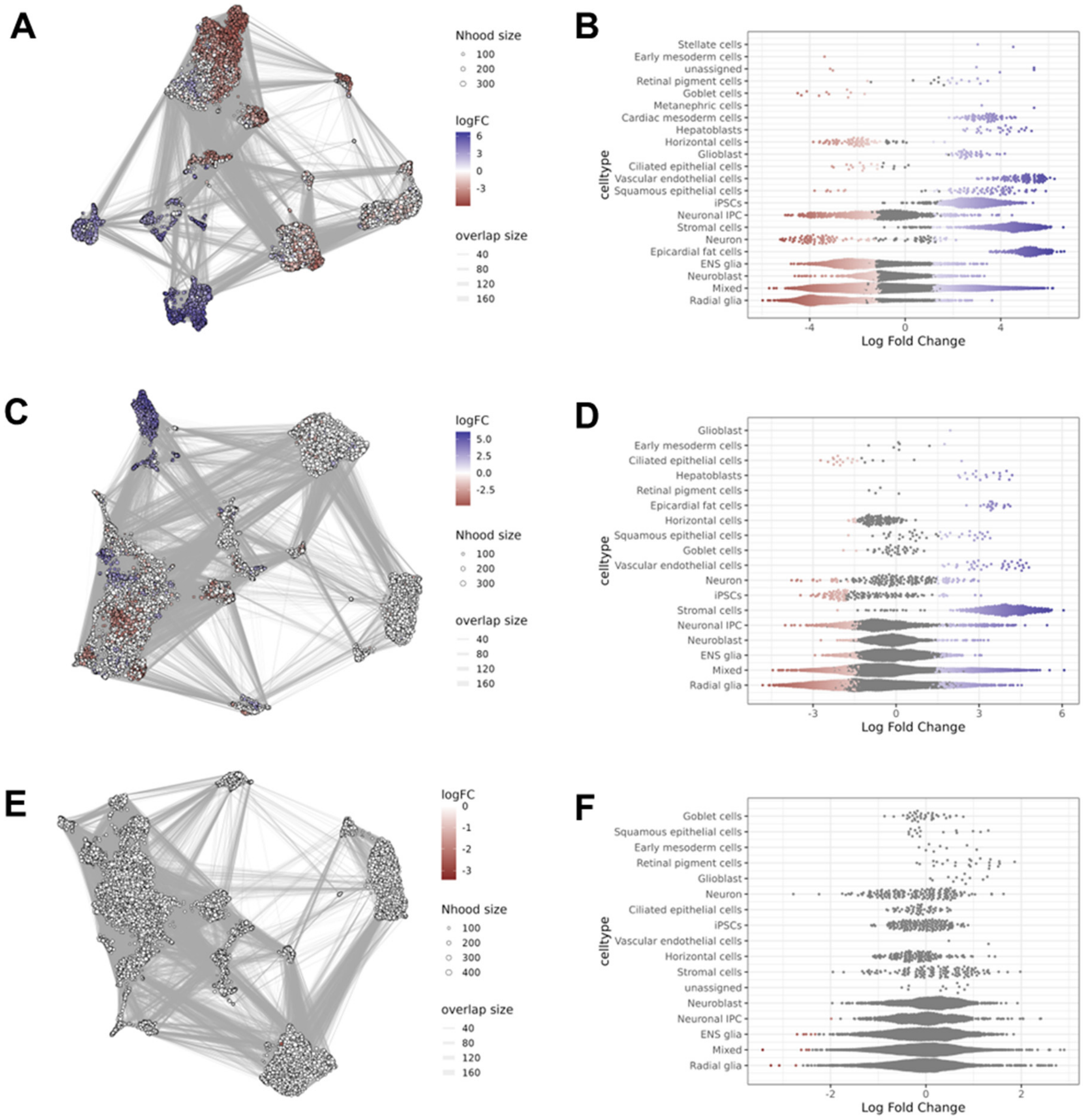

Figure S4. Differential abundance in response to treatment.
(A, C, E) Visualization of differential abundance on a KNN graph. Nodes are neighborhoods, colored by their log fold change between control and ethanol (A), caffeine (C), and nicotine (E), respectively. Non-differential abundance neighborhoods (FDR>5%) are colored white, and sizes correspond to the number of cells in each neighborhood. Graph edges depict the number of cells shared between neighborhoods
(B, D, F) Distribution of log fold change between treatment and control in neighborhoods containing cells from different cell type. Differential abundance neighborhoods at FDR<5% are colored. A neighborhood is assigned to a cell type if >75% cells within the neighborhood from the same predefined cell type.

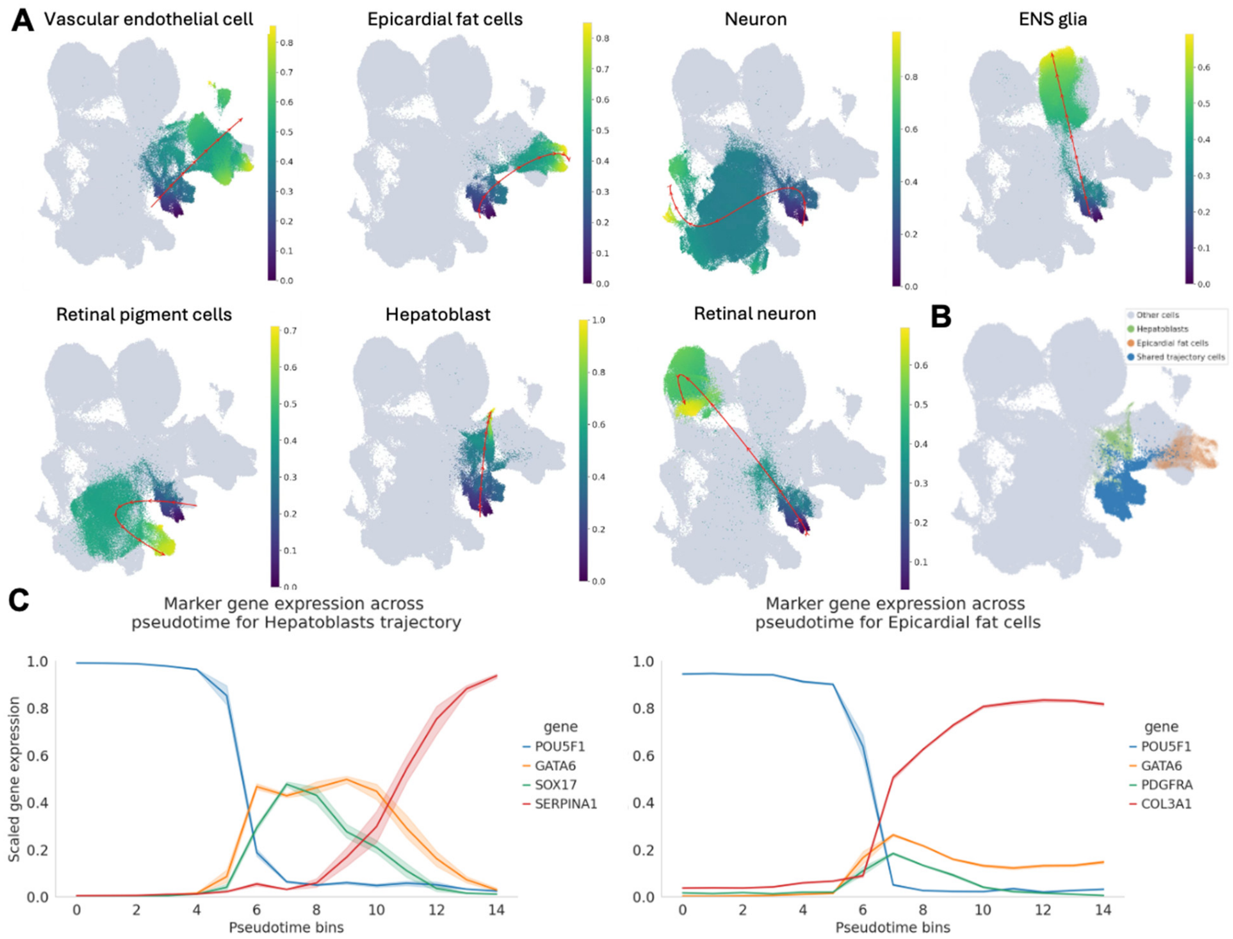

Figure S5. Full trajectories and trajectory validation.

(A) Pseudotime distributions for all inferred trajectories. Color bars represent pseudotimes of cells assigned to each trajectory. Cells not assigned to the trajectory are shown in gray.

(B) Hepatoblast and epicardial fat cell trajectories share intermediate mesendoderm cells. Cells assigned in hepatoblast only are shown in green, cells assigned in epicardial fat cells only are orange, and cells shared by the two trajectories are shown in blue. Cells not assigned to either trajectory are shown in gray.

(C) Marker gene expression for hepatoblast (left) and epicardial fat cell (right) trajectories. *POU5F1* is pluripotent cell marker gene, *GATA6* is the intermediate mesendoderm marker gene, *SOX17* is intermediate mesoderm marker gene, *PDGFRA* is intermediate endoderm marker gene, *SERPINA1* is hepatoblasts marker gene, and *COL3A1* is epicardial fat cell marker gene.

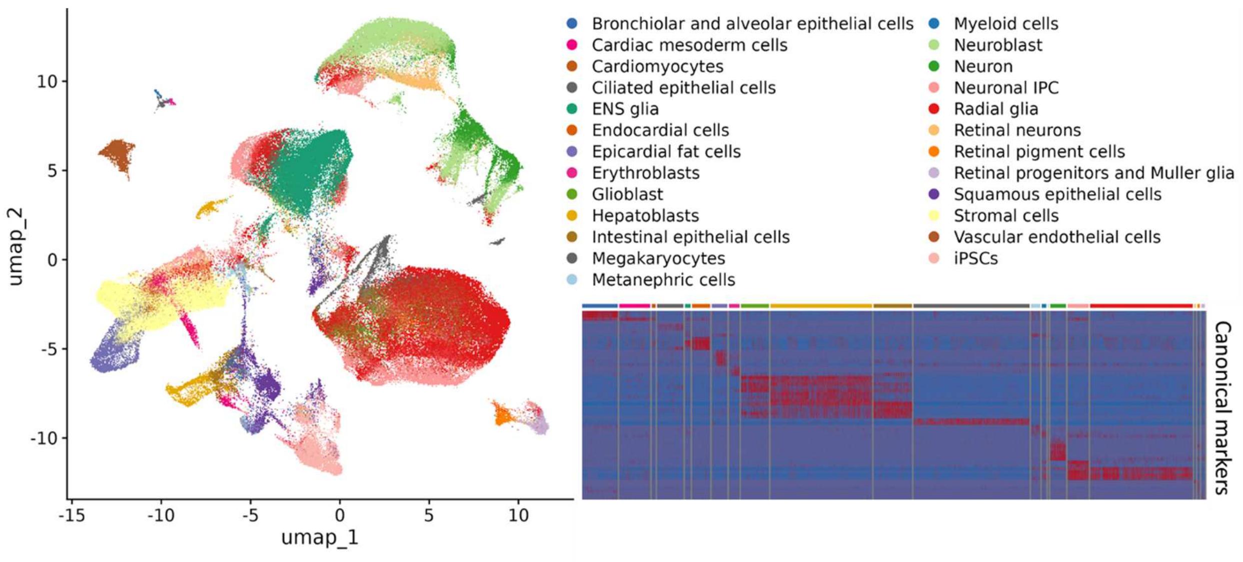

Figure S6. Validation of 25 high-confidence cell types.
UMAP visualization of cell types in different colors and heatmap of expression of canonical markers for each cell type.

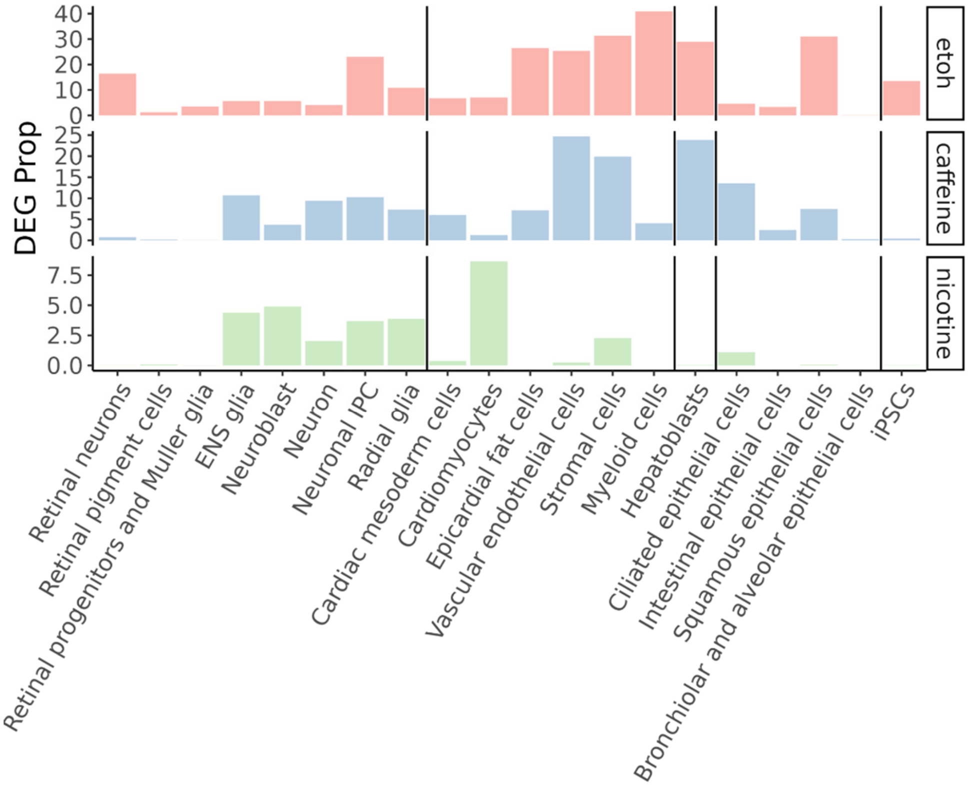

Figure S7. Proportion of treatment-induced differentially expressed genes in each cell type.

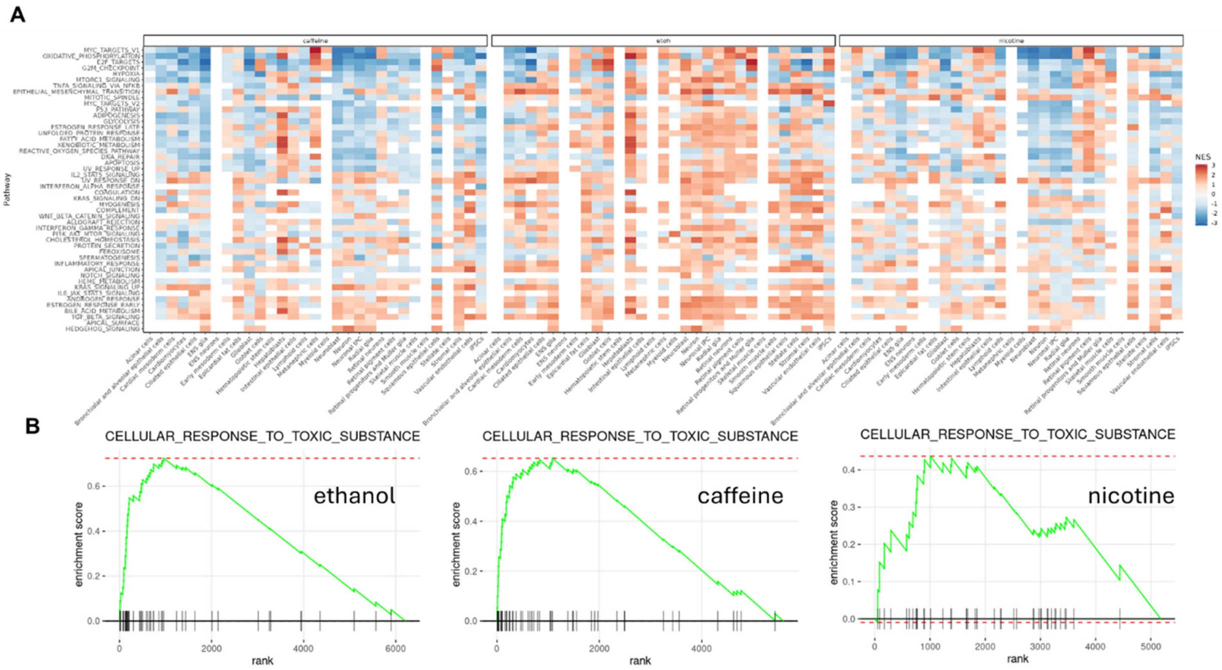

Figure S8. Pathway enrichment of differentially expressed genes in response to each treatment.
(A) Heatmap of enrichment in 50 hallmark gene set. Normalized enrichment score (NES) indicates direction and strength of enrichment, with enrichment in red and depletion in blue.
(B) Ranks of genes involved in cellular response to toxic substances by t-statistics of differential expression test in hepatoblasts in response to ethanol, caffeine, and nicotine, respectively.

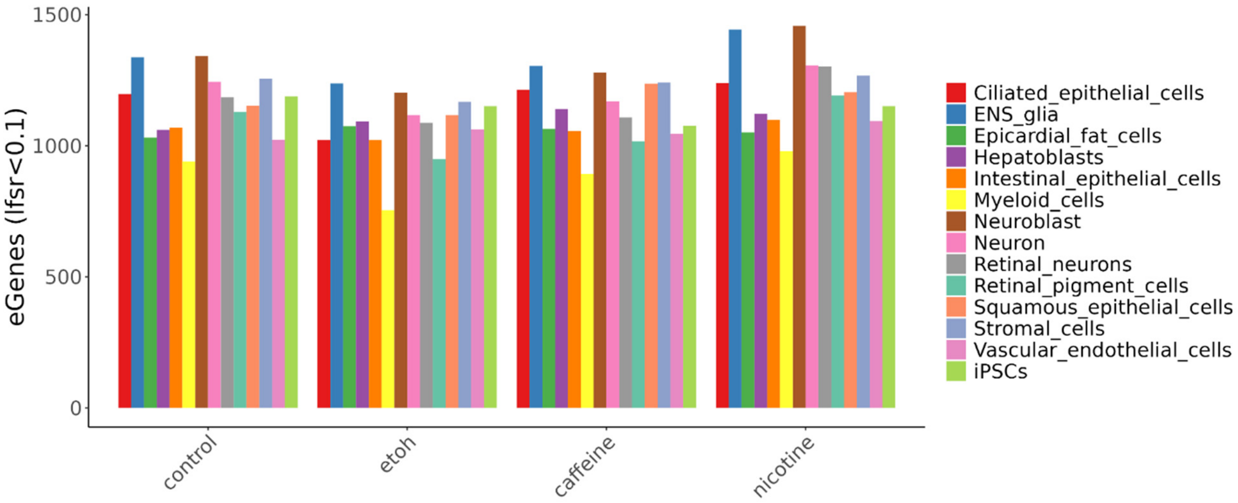

Figure S9. eGenes discovery in each cell type and condition.

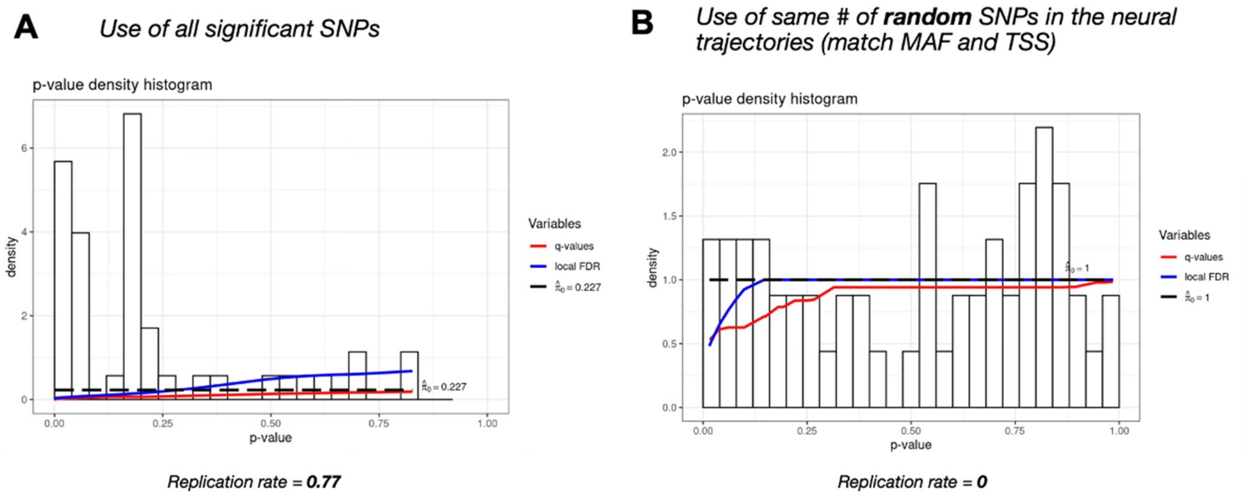

Figure S10. Replication of dynamic eQTLs.
(A) Replication of control neural dynamic eQTLs in J. Popp and K. Rhodes et al. and (B) their null set of eQTLs with matched minor allele frequence (MAF) and transcription start site (TSS).

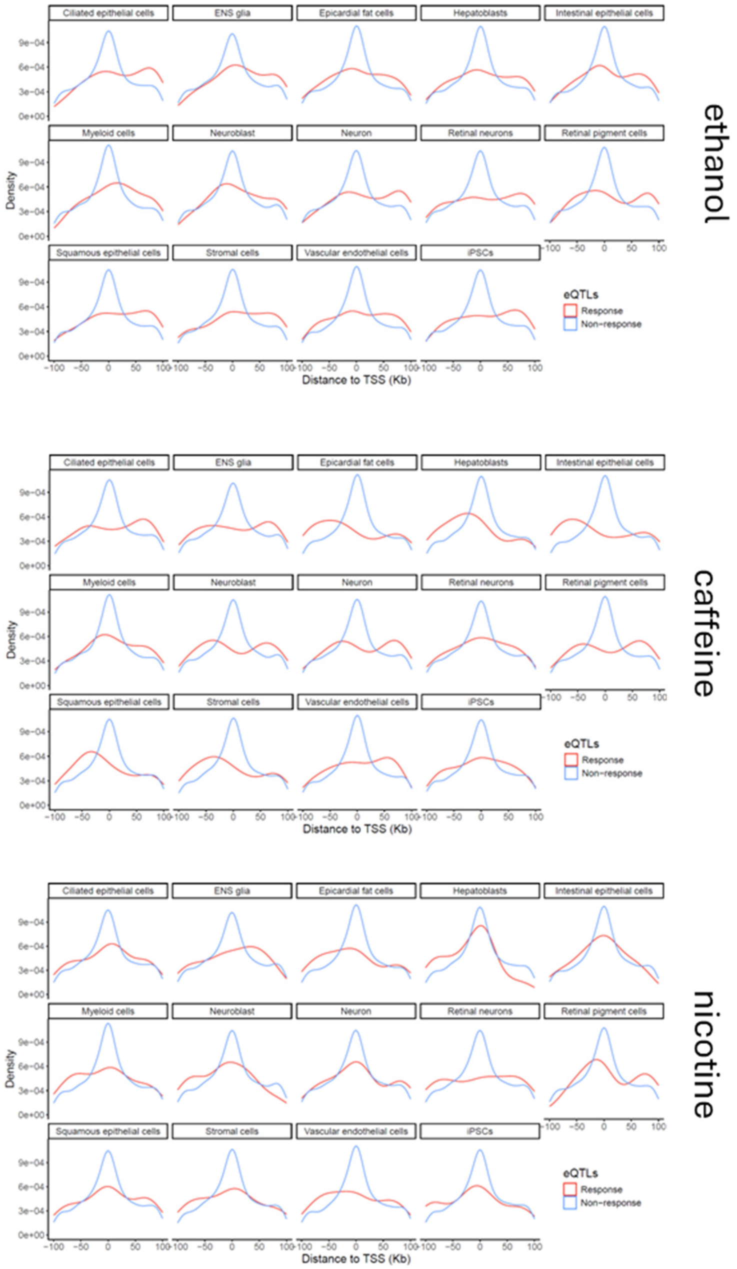

Figure S11. Distribution of distance to TSS for response eQTLs and non-response eQTLs in each cell type and treatment.

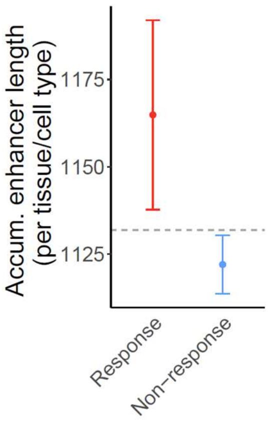

Figure S12. Average cumulative enhancer length per tissue/cell type.

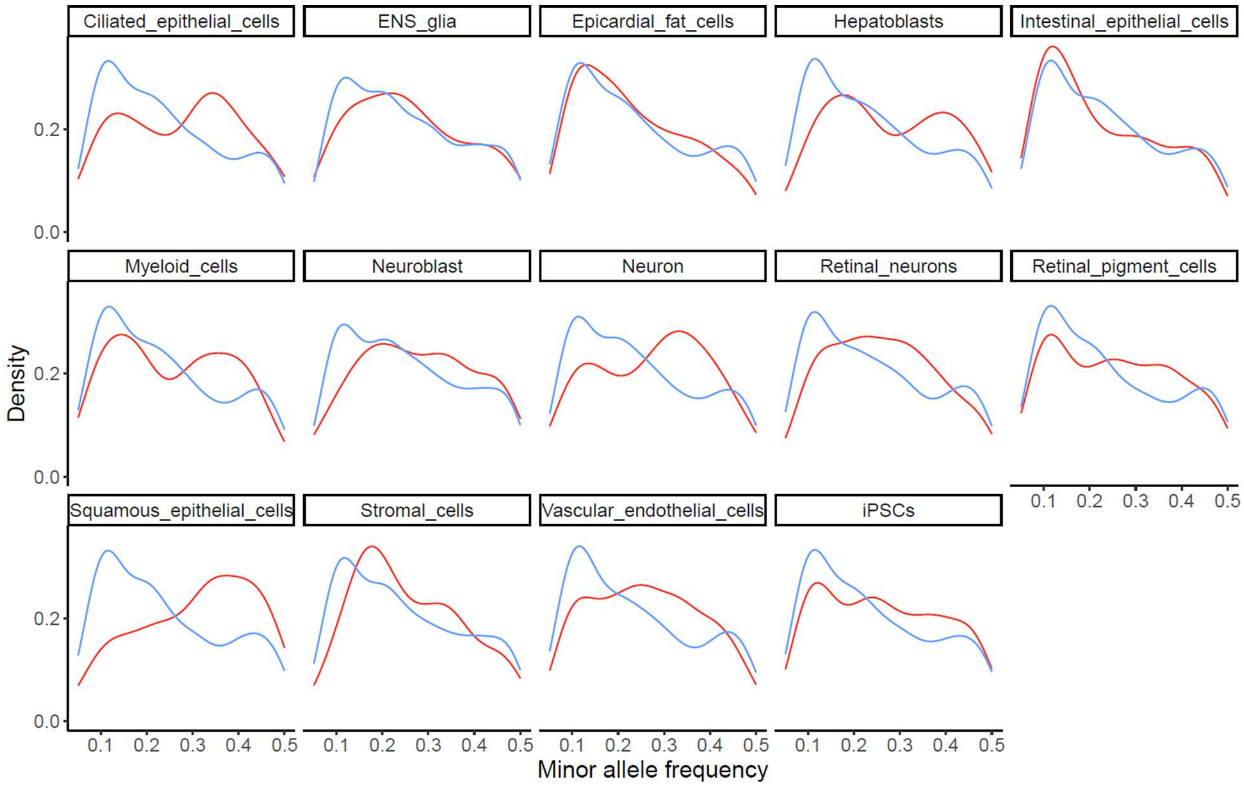

Figure S13. Distribution of minor allele frequency (MAF).

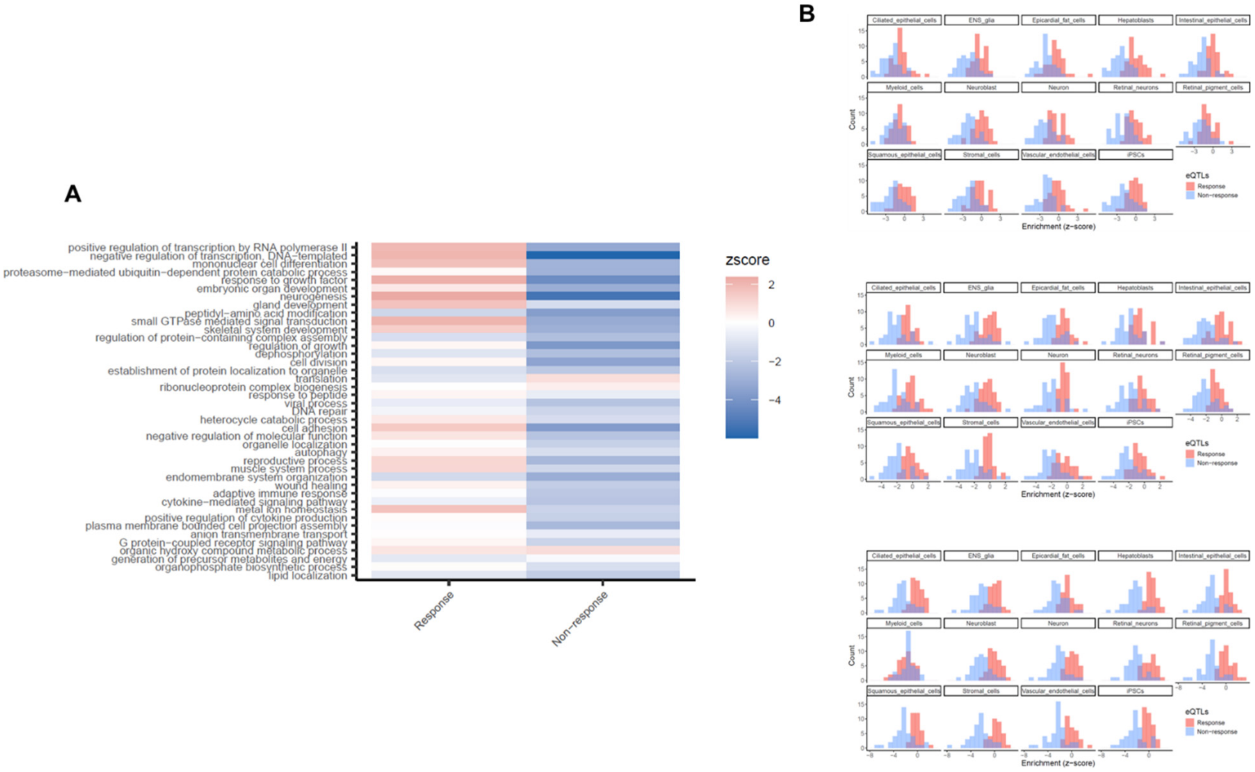

Figure S14. Enrichment of response eGenes and non-response eGenes in biological processes.
(A) Enrichment of response eGenes and non-response eGenes among genes in 41 broadly defined Gene Ontology (GO) categories. The GO categories (y-axis) are sorted based on the average pLI value of the corresponding genes within each category. The color map represents enrichment (red) or depletion (blue) by enrichment Z scores.
(B) Distribution of enrichment Z scores across 41 GO terms for response eGenes (red) and non-response eGenes (blue). From top to bottom: ethanol, caffeine, and nicotine.

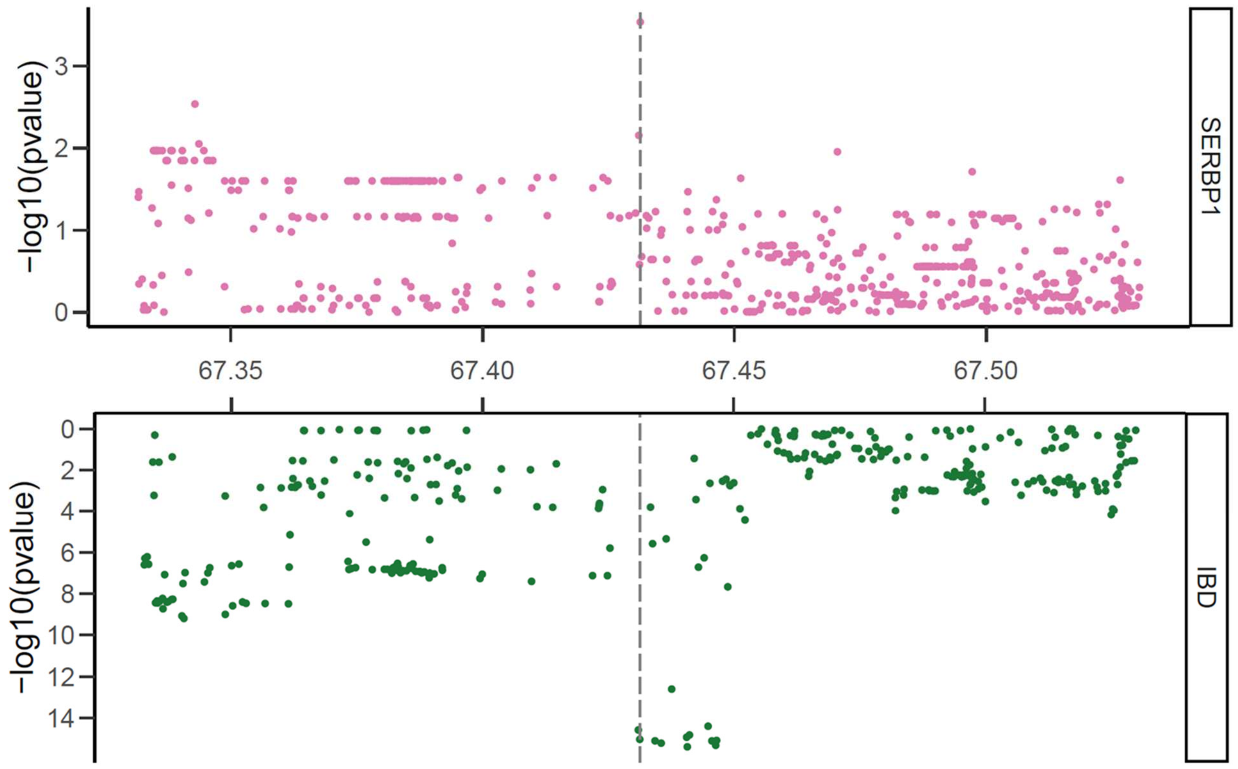

Figure S15. Colocalization of a response eQTL with IBD.
Visualization of an example of a caffeine-response eQTL (chr1:67431276:G_T) for *SERBP* in intestinal epithelial cells colocalized with a variant associated with IBD. The top panel (pink) shows significance levels of variants evaluated as eQTLs for the given gene expression including all variants within 100kb of the transcription start site, and the bottom panel (green) shows significance levels of variants tested for association with the trait(s) within the same region. Vertical lines depict the genomic location of the candidate colocalized variant.

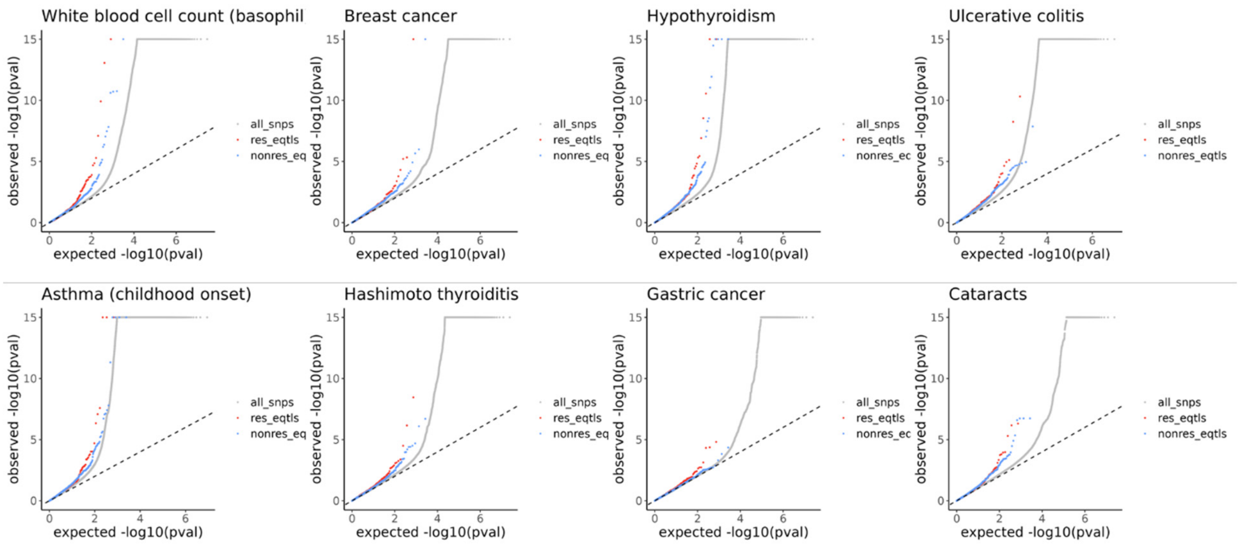

Figure S16. Examples of stronger enrichment of GWAS P-values in response eQTLs.

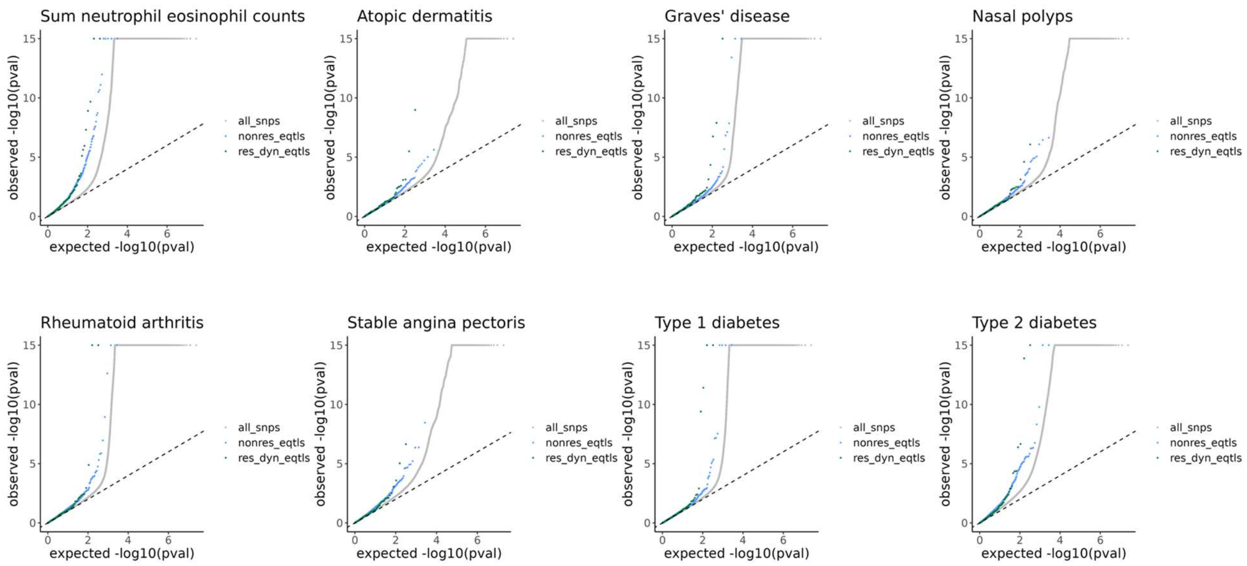

Figure S17. Examples of stronger enrichment of GWAS P-values in dynamic response eQTLs.

Supplementary Files

This is a list of supplementary files associated with this preprint. Click to download.

- TableS1cellculturemeta.xlsx
- TableS2referencemarkerlistforcellannotation.csv
- TableS3sctrajectoryassignment.csv
- TableS4perturbationinduceddifferentiallyexpressedgenes.csv
- TableS5responseeqtls.csv
- TableS6dynamiceqlts.csv
- TableS7colocresall.csv
